# Impact of CMV latency on T-cell responses to COVID-19 vaccination among predominantly antibody-deficient patients

**DOI:** 10.3389/fimmu.2025.1659259

**Published:** 2025-09-30

**Authors:** Trinidad Alba-Cano, Roberto Alonso, Héctor Balastegui-Martín, Luz Yadira Bravo-Gallego, Paloma Sánchez-Mateos, Mónica Martín-López, Juana Gil-Herrera

**Affiliations:** ^1^ Division of Immunology, Hospital General Universitario “Gregorio Marañón”, Madrid, Spain; ^2^ Department of Immunology, Ophthalmology and Otolaryngology, School of Medicine, Universidad Complutense, Madrid, Spain; ^3^ Department of Clinical Microbiology and Infectious Diseases, Hospital General Universitario Gregorio Marañón and CIBER (Centro de Investigación Biomédicas en Red) de Enfermedades Respiratorias, CIBERES, Barcelona, Spain; ^4^ Department of Medicine (Microbiology), School of Medicine, Universidad Complutense, Madrid, Spain; ^5^ Instituto de Investigación Sanitaria Gregorio Marañón (IiSGM), Madrid, Spain; ^6^ Center for Biomedical Network Research on Rare Diseases (CIBERER U767), Madrid, Spain; ^7^ Clinical Immunology Department, La Paz University Hospital, Madrid, Spain; ^8^ Research on Comprehensive Care for Transplanted Children and Adolescent Group, La Paz Institute for Health Research (IdiPAZ), Madrid, Spain

**Keywords:** COVID-19, SARS-CoV-2, mRNA vaccines, inborn errors of immunity, IEI, common variable immunodeficiency, CVID, CMV

## Abstract

**Background:**

The immunogenicity of mRNA COVID-19 vaccines has been reported as highly variable in patients with inborn errors of immunity (IEI).

**Objective:**

The aim of this study was to study memory CD4^+^ T-cell-mediated responses against the Spike (S) protein of SARS-CoV-2 along with CMV peptides in a large IEI group composed of mostly predominantly antibody-deficient (PAD) patients.

**Patients and methods:**

*In vitro* antigen-specific T-cell anti-S and -CMV responses after two doses of mRNA COVID-19 vaccines were assessed in peripheral blood from 114 patients with IEI and 38 healthcare healthy controls (HCHC). Stimulation index (SI) based on the percentages of CD4^+^ T lymphocytes with effector memory phenotype CD45RA^−^CD27^−^ (TEM) was quantified by flow cytometry.

**Results:**

Patients with IEI overall, as well as the two main groups of PAD [i.e., common variable immunodeficiency (CVID) and isotype or functional antibody deficiencies (IOFD)], showed frequencies of responder individuals and median SI against SARS-CoV-2 comparable to HCHC. However, those IEI and CVID subgroups positive for anti-CMV T-cell immunity showed a significantly reduced response (SI) against S-peptides when compared to their IEI and CVID counterparts who were anti-CMV TEM negative. This effect of CMV stratification is independent of age in our patient group.

**Conclusion:**

CMV latency negatively impacted the CD4^+^ TEM population’s functionality regarding COVID-19 vaccination in patients with CVID. Our results in patients with IEI and previous similar findings in healthy populations highlight the fact that when assessing immune-specific responses, the inclusion of CMV monitoring is suitable, is worthwhile, and may potentially be extended to vaccinations against different pathogens to prevent human disease more accurately.

## Introduction

SARS-CoV-2 has been the most recent infectious challenge worldwide, and huge efforts were made to combat the COVID-19 pandemic. SARS-CoV-2 vaccines received emergency approval and became the main effective measure to reduce the incidence of cases, hospitalizations, and mortality in the general population globally (1, 2).

When the clinical benefits of COVID-19 vaccines have been studied in patients with inborn errors of immunity (IEI) (formerly called primary immunodeficiencies), reduced inpatient and intensive care unit (ICU) admissions and mortality were also demonstrated in these higher-risk patients, although worse outcomes remain superior compared to the general population (3–5).

Regarding COVID-19 vaccines’ immunogenicity, very variable seroconversion rates, antibody titers, and T-cell responses have been detected across the different IEI categories so far (6–9), highlighting the huge IEI heterogeneity and pointing to the idea that cellular response may be more important than humoral response in such COVID-19-vaccinated patients.

Several conditions such as age, concomitant use of immunosuppressive medication, and infections and non-infectious related complications may also underlie some previous controversial results. Within the group of predominantly antibody deficiencies (PADs), very interesting studies in patients with common variable immunodeficiency (CVID) have demonstrated that the presence of autoimmune cytopenia, lymphoproliferative disorders, and granulomatous-lymphocytic interstitial lung disease can influence the immunogenicity of SARS-CoV-2 vaccines (10, 11).

The evaluation of specific T-cell responses after SARS-CoV-2 infection and vaccination in the context of IEI stands to reason given the critical role of T lymphocytes in the control and clearance of viral human diseases (12, 13). Moreover, in COVID-19, T-cell-mediated immunity confers a diverse and broadly reactive immune response to ancestral and emerging SARS-CoV-2 variants of concern (14, 15). As most patients with IEI suffer from PAD, T-cell immunity reflects their real ability to generate immune responses to COVID-19 vaccines much better than antibodies. In many of these PAD patients, the quantitation of anti-SARS-CoV-2 immunoglobulin G (IgG) may be overrepresented due to its presence in the immunoglobulin preparations used as replacement therapy, or underrepresented because of the hampered production of antigen-specific antibodies while they are not being treated with immunoglobulin replacement therapy (IgRT) (16).

Cytomegalovirus (CMV) is not a new but, rather, a very old DNA pathogen belonging to the family *Herpesviridae*, which is considered a modulator of the immune system function, largely due to the induction of immunosenescence, memory inflation, and adaptation of the immune repertoire (17–22). In healthy CMV-seropositive individuals, a high proportion of the peripheral circulating T-cell pool is CMV-specific (23), with expansion of effector-memory T cells and a marked decrease in naïve T-cell subsets (24, 25).

Human latent CMV infection has been linked to more severe outcomes of other infectious diseases due to a failure to control different viral infections (20). Regarding COVID-19, worse clinical outcomes (26–28) and the development of long-COVID (20, 29, 30) have already been observed in CMV-seropositive individuals. Moreover, Aquino et al. found higher proportions of effector CD4^+^ T lymphocyte subsets in African populations—with a known superior prevalence of CMV (99% seropositive individuals)—when compared to Europeans, and suggested that these differences may affect immune responses to SARS-CoV-2 infection (31).

The impact of latent CMV infection on SARS-CoV-2 T-cell immunity following COVID-19 vaccination was first described in 2024, in healthy Dutch adults (32). This important work has demonstrated that adults aged 70 years or above showed decreased SARS-CoV-2-specific interferon-γ (IFN-γ) responses compared to younger age groups, but only in the CMV-seropositive cohorts (32). On the other hand, no significant differences had been found previously in ChAdOx1 nCoV-19 vaccine-specific T-cell responses when healthy young adult volunteers (aged 18–55 years) were stratified by CMV serostatus (33). In another cohort of healthy individuals, the longevity of memory—cellular or humoral—response after SARS-CoV-2 mRNA vaccination was not decreased in CMV-seropositive older adults (≥65 years) when compared to those who were seronegative (34).

We recently reported on a patient with CD4^+^ lymphopenia who showed persistent and strong T-cell responses to CMV but an impaired ability to mount T-cell or antibody responses to SARS-CoV-2 throughout five immunizations (35), which led us to consider CMV infection as a potential additional risk factor for a reduced immunogenicity of COVID-19 vaccines in other immunodeficient patients.

To the best of our knowledge, no reports have been published that investigate a relationship between previous exposure to CMV and vaccine-mediated T-cell immunity against SARS-CoV-2 in patients with IEI. Our aim was to assess T-cell responses to SARS-CoV-2 vaccination and also evaluate whether CMV latency could influence T-cell immunogenicity of mRNA COVID-19 vaccines in a large group of patients with IEI.

## Patients and methods

### Study groups

Our single-center cross-sectional observational study enrolled 114 patients with IEI belonging to the National Reference Unit for Immunodeficiency at the Gregorio Marañón University Hospital in Madrid (Spain). There were 69 women and 45 men, aged between 22 and 80 years [median (interquartile range): 54 (44–63) years]. Between February and September of 2021, 98 out of the 114 patients with IEI (86%) received two doses of the mRNA-1273 COVID-19 vaccine (Moderna, Cambridge, MA, USA) 28 days apart, and the remaining 16 patients (14%) received two doses of the BNT162b2 COVID-19 vaccine (Pfizer, New York, NY, USA) with an interval of 21 days. Peripheral blood samples for assessing immune responses to SARS-CoV-2 and CMV were collected with a median of 89 (72–120) days after the second dose of vaccination.

Most of the patients with IEI (103) were naïve to SARS-CoV-2 infection according to the clinical information regarding COVID-19 derived from their medical records throughout the close follow-up, which was performed in our Outpatient Clinical Unit and Day Hospital from the beginning of the pandemic. At the time of the study, only 11 patients were infected with SARS-CoV-2 before the administration of mRNA COVID-19 vaccine and just 1 patient was infected after the two vaccine doses but before the evaluation of immune response. Infection was documented by positive nasal swab SARS-CoV-2 polymerase chain reaction (PCR) tests in four patients and antigen rapid tests in three patients. In the remaining five patients, positive SARS-CoV-2 IgG serology was detected before vaccination. Although three out of these five patients were under IgRT, IgG anti-SARS-CoV-2 should not have been passively transferred by immunoglobulin substitution but produced by the patients, since the serum samples of the three of them were obtained before April 2021 and immunoglobulin products did not contain SARS-CoV-2-specific antibodies until late 2021 or early 2022 (36).

A total of 38 healthcare workers were also included as healthy controls (HCHC). Thirty of them were women and eight were men, aged between 26 and 65 years [50 (31–59)]. Of the 38 healthcare workers, 34 (89%) were vaccinated with two doses of BNT162b2 vaccine and 4 (11%) received primary mRNA-1273 vaccination between January and July of 2021. Peripheral blood samples were collected within 118 to 226 (median, 158) days after primary vaccination. All HCHC individuals were naïve to SARS-CoV-2 infection as known by repeated negative results of PCR routinely assessed by the Division of Labour Risks Prevention of our Hospital.

No severe adverse side effects of the mRNA vaccines were detected in either group.

This study was carried out in accordance with the Declaration of Helsinki and approved by the Ethics Committee (IEI vac SARS-CoV-2) of the Gregorio Marañón University Hospital.

### Evaluation of virus-specific CD4^+^ T-cell immunity: AIM assay

An activation-induced markers (AIM) assay was used to evaluate *in vitro* virus-specific CD4^+^ T-cell immunity by multicolor flow cytometry as described previously (35).

Peripheral blood mononuclear cells (PBMCs) were isolated by density gradient and resuspended in a concentration of 10 × 10^6^ cells/mL in TExMACS medium with stable glutamine (Miltenyi Biotec, Bergisch Galdbac, Germany) and supplemented with 10% fetal bovine serum. A total of 1 × 10^6^ cells per well were plated in 96-well U-bottom plates (Corning Inc., New York, NY, USA) along with 1 μL of co-stimulatory monoclonal antibodies CD28/CD49d [clones L293/L25] for each condition. Under specific antigen conditions, 2 μL of peptide pool compounds of 15-mer peptides with 11-amino-acid overlap was included. In case of CMV, the peptide pool covered the complete sequence of the pp65 protein (Peptivator CMV pp65, premium grade, Miltenyi Biotec, Bergisch Galdbac, Germany). For SARS-CoV-2, it covered the original strain of spike protein (Peptivator SARS-CoV-2 Prot_S, Miltenyi Biotec, Bergisch Galdbac, Germany). As a positive condition, 4 μL of anti-human CD3/CD28/CD2 antibodies (ImmunoCult™ Human CD3/CD28/CD2 T cell Activator, STEMCELL Technologies, Vancouver, BC, Canada) was added. Negative control condition was supplemented with 2 µL of medium. Then, cells were incubated for 44–48 h at 37°C in a humidified atmosphere containing 5% CO_2_.

Following incubation, cells from each well were recollected separately in flow cytometry single tubes and washed with 1 mL of phosphate-buffered saline (PBS)/EDTA buffer. After that, the cells in every tube were stained with 20 μL of a commercial kit of CD4 T-cell activation (Act-T4 Cell (CYT-AT4C) antibody combination (BD Biosciences, San Jose, CA, USA) which contain a mix of monoclonal antibodies against CD3 PerCP-Cy5.5 [clone UCHT1] and CD4 FITC [clone RPA-T4]; and CD25 APC [clone M-A251] and OX40 (CD134) PE [clone 134-1] as AIM. Moreover, 1 μL of CD45RA APC-H7 [clone HI100] and 1 μL of CD27 PE-Cy7 [clone M-T271] monoclonal antibodies were included to identify effector memory subsets of CD4^+^ T cells. Additionally, 3 μL of PBS was added to every tube. All monoclonal antibodies were from BD Biosciences. Cells were incubated for 20 min at room temperature in the dark, washed again, and resuspended in 200 μL of PBS buffer. Finally, stained cells were acquired using a FACSLyric cytometer from BD Biosciences, and at least 100,000 lymphocytes were recorded per sample. Flow cytometry data were analyzed with BD FACSuite software version 1.5.

Gating strategy is shown in **Supplementary Figure S1A**. Lymphocytes were gated based on size and granularity characteristics. Then, CD4^+^ T cells were selected by CD3 and CD4 expression and further classified by surface differentiation markers CD45RA and CD27 into effector memory (TEM) helper T cells (CD3^+^CD4^+^CD45RA^−^CD27^−^). Cellular immune response was expressed as the stimulation index (SI) calculated by dividing the percentage of CD4^+^ TEM AIM^+^ cells [co-expressing OX40 (CD134) and CD25] in stimulation conditions by the percentage of CD4^+^ TEM AIM^+^ cells in negative control. SI ≥ 2 was considered positive (35). Four representative examples are shown in **Supplementary Figure S1B**.

### Statistics

GraphPad Prism version 10 (GraphPad Software, San Diego, CA) was used to perform statistical analysis and graphical representation of the data. Data normality was previously checked in all variables using the Kolmogorov–Smirnov test. Differences between number of responder individuals (categorical variables) were assessed using Fisher’s exact test and were expressed in frequency or percentage along with their corresponding 95% confidence intervals (CIs) calculated using the Wilson/Brown method. Differences between the SI of antigen-specific CD4^+^ T-cell responses (continuous variables) were compared by the non-parametric Mann–Whitney *U* test for non-paired samples and were reported as median and interquartile range (IQR = P25–P75) and 95% CIs. A multiple linear regression analysis was performed to evaluate the contribution of latent CMV infection (defined as the positive/negative T-cell response to CMV status) and the age (in years) as independent variables on the cellular quantitative response to SARS-CoV-2 as the dependent variable. In each statistical graph, horizontal bars represent the median and IQR values. *p* < 0.05 was considered significant (two-sided) and the significance is depicted with the *p*-value.

## Results

**Table 1** shows the classification of our IEI group according to the IUIS criteria (37): 105 were categorized as PAD [48 had CVID, 52 had isotype chain or functional antibody deficiencies (IOFD), 3 had agammaglobulinemia, and 2 had hyper-IgM syndromes], 2 patients had diseases of immune dysregulation, 3 had combined immunodeficiencies (2 di George and 1 Good syndromes), 1 was diagnosed as having a phagocytic disorder, and 3 had complement deficiencies. As shown in **Table 1**, 60/114 patients with IEI were receiving periodic intravenous or subcutaneous IgRT.

**Table 1:** Characteristics of patients and healthy controls.

Group	*n*	Median age (years)	Sex (male/female)	IgRT (iv/sc)	Vaccine (Moderna/Pfizer)	Median days since second dose to blood collection
Patients with IEI (overall)	114	54 (n.s.)	45/69 (*)	42/18	98/16	89
Predominantly antibody deficiency	105	54	42/63	40/18	92/13	91
• Common variable immunodeficiency	48	52	20/28	26/17	44/4	86
• Isotype, light chain, or functional deficiencies	52	57	19/33	12/0	44/8	104
• Agammaglobulinemia	3	49	3/0	2/1	2/1	62
• Hyper IgM syndromes	2	56	0/2	0/0	2/0	54
Combined immunodeficiencies syndromic features	3	26	2/1	1/0	2/1	60
Complement deficiencies	3	50	1/2	0/0	1/2	89
Diseases of immune dysregulation	2	58	0/2	1/0	2/0	32
Congenital defects of phagocytes	1	62	0/1	0/0	1/0	97
HCHC	38	50	8/30	–	4/34	158

IgRT, immunoglobulin replacement therapy; iv, intravenous; sc, subcutaneous; IEI, inborn error of immunity; HCHC, healthcare healthy controls; n.s., not statistically significant. *p < 0.05.

No significant difference was found in the median age of our patients with IEI when compared to HCHC. Regarding sex, the proportion of female patients was significantly higher (*p* = 0.0492) in the HCHC group than in the group of patients with IEI.

**Figure 1:**
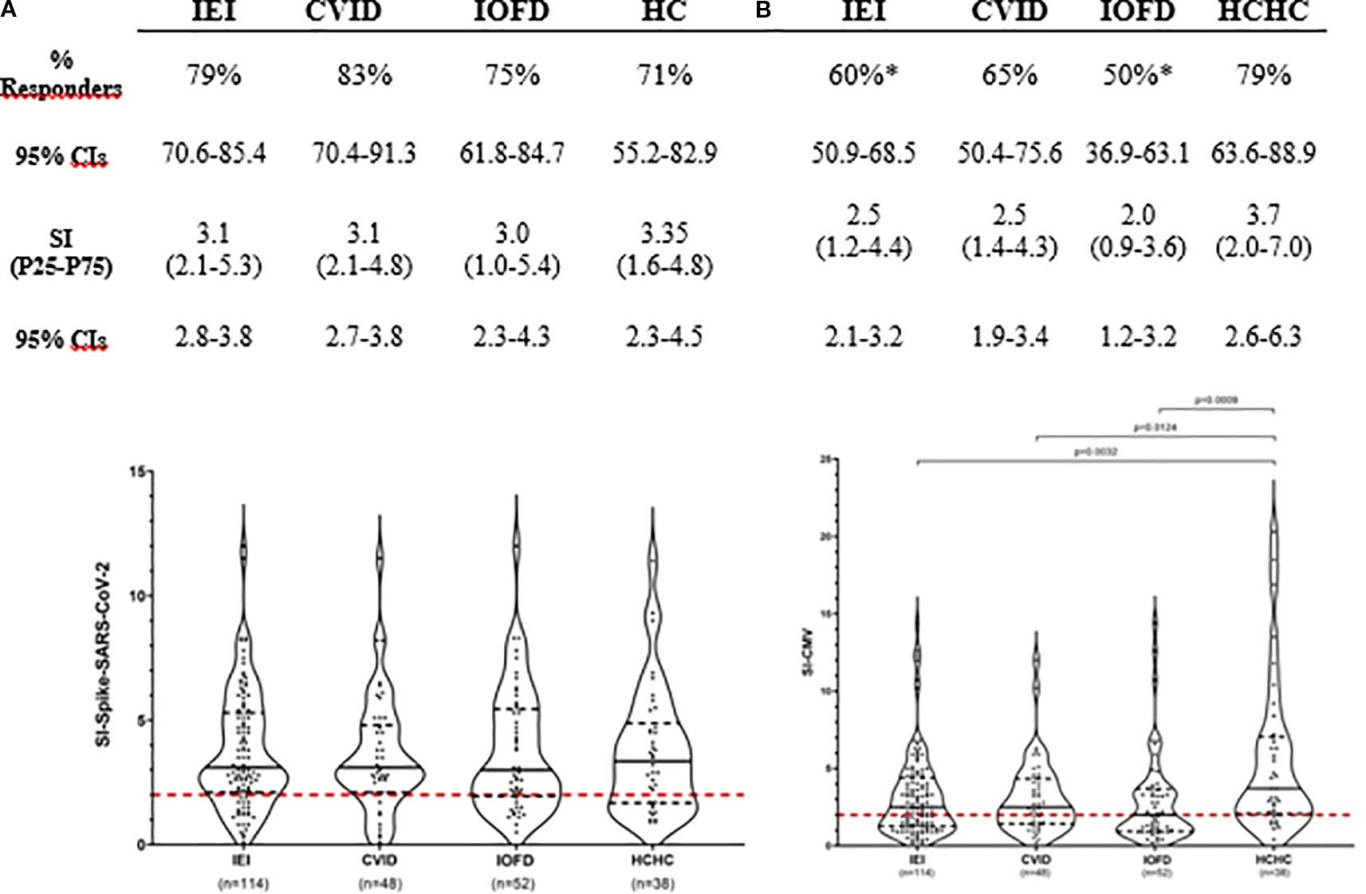
Simultaneous assessment of T-cell immunogenicity after two doses of COVID-19 vaccination and CMV-specific T-cell reactivity in patients with IEI and healthy controls. Responders’ frequency (%) and magnitude (SI) of CD4^+^ TEM responses are shown for all patients with IEI, CVID and IOFD groups, and HCHC. Only those frequencies that according to Fisher’s exact test were significantly different when compared to the HC group are pointed with a superscript asterisk (*). Within the violin plots, only significant SI comparisons with *p* < 0.05 values are depicted, calculated by Mann–Whitney tests. Continuous black lines represent the median; the interquartile range is depicted by discontinuous black lines; discontinuous red lines represent the cutoff (SI ≥ 2) value for positivity. CVID, common variable immunodeficiency; HCHC, healthcare healthy controls; IEI, inborn error of immunity; IOFD, isotype or functional deficiencies; SI, stimulation index; CIs, confidence intervals; TEM, effector memory CD4^+^ T cells.

**Figure 1A** shows that 79% (90 out of the 114 patients with IEI) had circulating CD4^+^ TEM reactive to SARS-CoV-2 S-peptides. This percentage of responder patients and the median SI after S-antigen stimulation from the entire IEI group were not statistically significant when compared to the frequency of 71% (27 out of 38) and the intensity of T-cell response found in the HCHC group. Among the 24 non-responder patients with IEI (21%) who lacked circulating SARS-CoV-2 S-specific CD4^+^ TEM, 91.6% belonged to the PAD group. In order to analyze the potential impact of demographic differences on SARS-CoV-2 T-cell responses, we stratified IEI or HCHC groups by sex, but no significant differences were found in either the proportion of responders or their SI median values (not shown). When those 100 PAD patients from our IEI group were further classified as CVID or IOFD (**Figure 1A**), statistically significant differences were neither found in the frequency and intensity of CD4^+^ T-cell responses to S-peptides when compared to HCHC, or when comparing these two PAD major subgroups between them (i.e., CVID vs. IOFD).

Regarding CMV *in vitro* responses, as shown in **Figure 1B**, anti-CMV CD4^+^ TEM were detected in 68 out of the total 114 IEI participants (60%). The comparison of this frequency with 79% (30/38) found in HCHC reached statistical significance (*p* = 0.049), and the median SI of 2.5 against CMV peptides from all patients with IEI was also significantly lower (*p* = 0.0032) than the median SI in HCHC controls, which was 3.7. Both CVID and IOFD subgroups also showed a statistically significant reduced intensity of the CMV-specific CD4^+^ TEM response when compared to the SI from the HCHC group (median SI, 2.5 vs. 3.7, *p* = 0.01 and 2.0 vs. 3.7, *p* = 0.0009, respectively).

**Figure 2:**
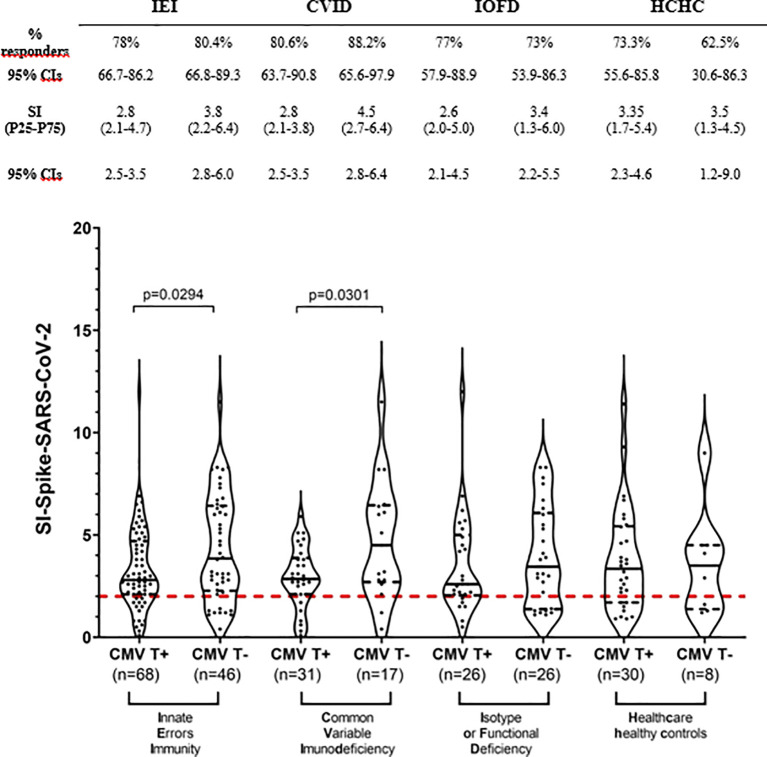
T-cell immunogenicity to two doses of COVID-19 vaccination in patients with IEI and healthy controls stratified by their CMV-specific T-cell status. Frequency (%) of responders and magnitude (SI) of T-cell responses to SARS-CoV-2 peptides in the study subgroups classified by positive or negative CD4^+^ TEM anti-CMV response. Only significant (*p* < 0.05) values are depicted, which correspond to median SI comparisons calculated by Mann–Whitney tests. Continuous black lines represent the median; the interquartile range is depicted by discontinuous black lines. Discontinuous red lines represent the cutoff (SI ≥2) positivity. CVID, common variable immunodeficiency; HCHC, healthcare healthy controls; IEI, inborn error of immunity; IOFD, isotype or functional deficiencies; SI, stimulation index; TEM, effector memory CD4^+^ T cells; CIs, confidence intervals.

Next, we stratified all IEI and HCHC individuals into CMV latently infected or non-infected groups, according to their *in vitro* CMV-specific T-cell reactivity (i.e., qualitative positive or negative anti-CMV CD4^+^ TEM). Although anti-CMV serology was tested in every patient and healthy control (not shown), we considered specific IgG uninformative since most PAD individuals weakly produce antibodies and/or they are under IgRT as reflected in **Table 1**. **Figure 2** shows that although the CMV-infected IEI patients’ subgroup had a similar frequency of SARS-CoV-2 responders (78%, 53/68) to the subgroup of patients with IEI lacking circulating anti-CMV CD4^+^ TEM (80.4%, 37/46), a statistically significant lower intensity in their SARS-CoV-2 responses (median SI, 2.8 vs. 3.8, *p* = 0.0294) was observed. This significant difference was replicated between the CVID group of patients, when comparing the median SI of 2.8 after SARS-CoV-2 *in vitro* stimulation from anti-CMV CD4^+^ TEM positive patients with CVID with the median SI of 4.5 in the patients with CVID who were anti-CMV CD4^+^ TEM negative. Such difference was not found when stratifying patients with IOFD or the HCHC group by their anti-CMV CD4^+^ TEM status, or in the frequency of responders to SARS-CoV-2 (**Figure 2**).

**Figure 3:**
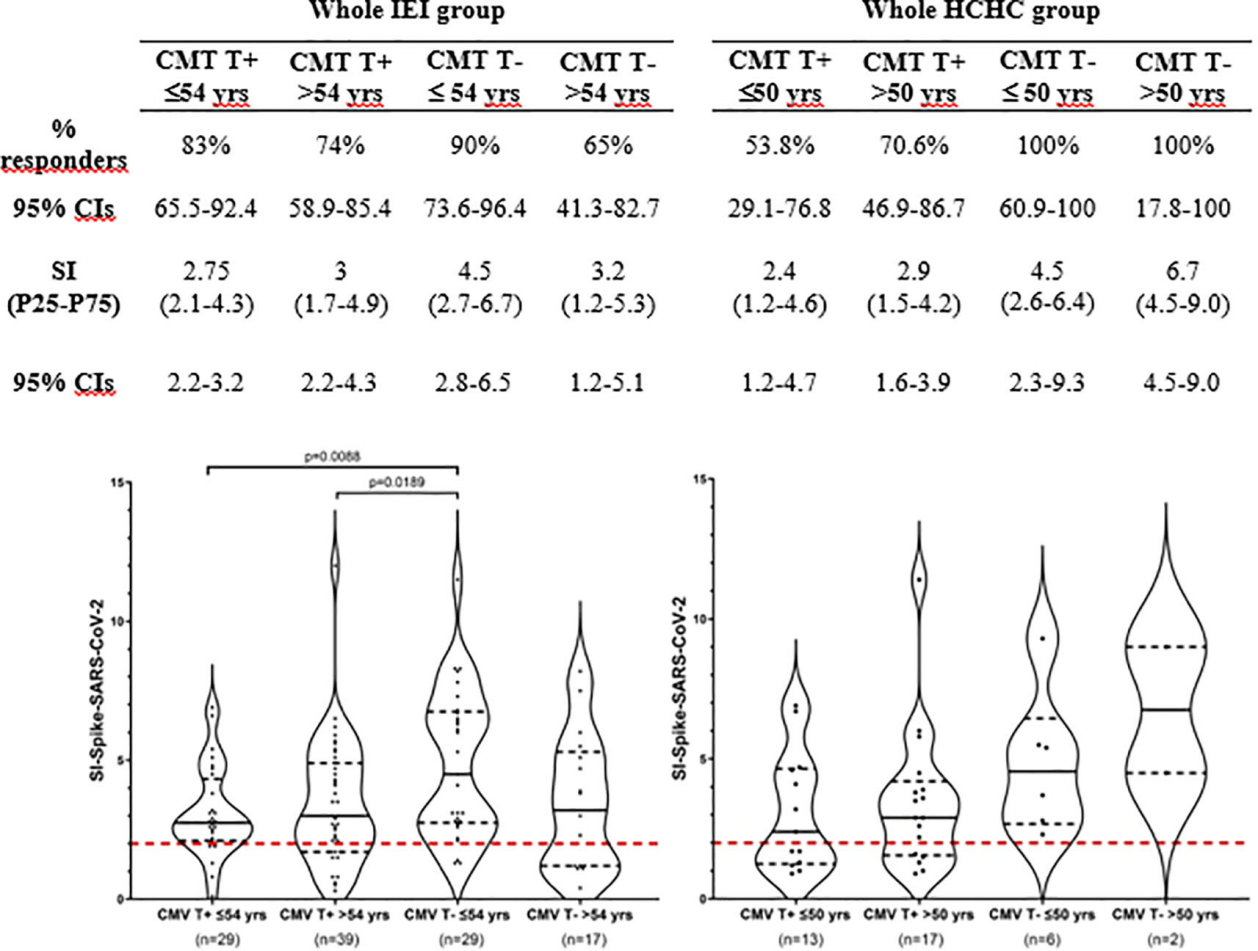
T-cell immunogenicity after two doses of COVID-19 vaccination following stratification by CMV-specific T-cell reactivity and age. Frequency (%) of responders and magnitude (SI) of T-cell responses to SARS-CoV-2 following two doses of COVID-19 vaccination in the entire group of patients with IEI and HCHC, categorized by positive or negative CD4^+^ TEM anti-CMV response and by the median age value of each group (54 years for IEI and 50 years for HCHC). Only significant (*p* < 0.05) values are depicted, which correspond to SI comparisons calculated by Mann–Whitney tests. Continuous black lines represent the median; the interquartile range is depicted by discontinuous black lines. Discontinuous red lines represent the cutoff (SI >2) positivity. HCHC, healthcare healthy controls; IEI, inborn error of immunity; SI, stimulation index; TEM, effector memory CD4^+^ T cells; CIs, confidence intervals.

To better delineate the contribution of CMV latent infection to impaired T-cell immunity against SARS-CoV-2, patients with IEI divided into two groups based on their T-cell reactivity to CMV were further stratified by age using the median age value (54 years old) of the entire IEI group. The patients with IEI with circulating anti-CMV CD4^+^ TEM (*n* = 68, mean age 56 ± 13.6 years) turned out to be significantly older (*p* < 0.005) than those who are anti-CMV CD4^+^ TEM negative (*n* = 46, 48 ± 13.1 years). **Figure 3** shows no apparent effect of age on COVID-19 vaccination-induced SARS-CoV-2-specific CD4^+^ TEM response when our group of patients underwent this analysis (i.e., patients with IEI with or without circulating anti-CMV CD4^+^ TEM, re-stratified as younger or older than 54 years). As expected, the highest SARS-CoV-2 CD4^+^ TEM response was found in the subgroup of CMV non-infected younger patients, but it is significant only when compared to both the younger and older subgroups of CMV latently infected patients with IEI, and non-significant when compared to its CMV counterpart of non-infected and older patients.

Finally, we performed a multiple regression analysis to determine the effect of CMV status and age and investigated their confounding interactions following SARS-CoV-2 vaccination. **Table 2** shows the results of the multivariant model, confirming that the significant reduction of *in vitro* specific CD4^+^ TEM response against SARS-CoV-2 within patients with latent CMV infection is independent of age in our COVID-19-vaccinated patients with IEI.

**Table 2:** Linear regression model for SARS-CoV-2 T-cell responses after two doses of mRNA COVID-19 vaccination in all patients with IEI.

Variable	Estimate	Standard error	*p*-value
Intercept	4.792	0.829	<0.0001
CMV	−1.032	0.451	0.024
Age	−0.009	0.015	0.577

The dependent variable is the magnitude of TEM anti-SARS-CoV-2 response measured by the stimulation index. Independent variables are qualitative anti-CMV TEM response and age in years. A significance level of *p* < 0.05 was considered for all two-tailed tests.

## Discussion

This study shows, for the first time, CD4^+^ T-cell immune responses against SARS-CoV-2 and CMV when tested simultaneously by using a single-cell, multicolor flow cytometry-based assay. The COVID-19 pandemic challenged many clinical and research immunology laboratories to implement diverse *in vitro* SARS-CoV-2 antigen-specific T-cell assays; an AIM platform enabled us to perform immunophenotyping and gating of responder memory T lymphocytes, which is considered highly sensitive and specific (38–40).

Although the utility of tests measuring CMV-specific T-cell immunity has not yet been established in IEI cohorts (41), such laboratory assays are currently used for the clinical management of other immunocompromised patients (i.e., patients undergoing solid organ and hematopoietic precursor cell transplantation) (42, 43). In our study design, CMV peptides were included as a control for every laboratory assay (44) as well as a different viral stimulation for a better understanding of the CMV immune status of our patients with IEI (35). This approach finally allowed us to study the impact of CMV infection on the T-cell immunogenicity of SARS-CoV-2 vaccination in our IEI group.

According to exhaustive reviews on humoral and T-cell immunity after two doses of SARS-CoV-2 vaccines, 30% to 87% of patients with IEI showed vaccine-specific T cells (6–9). Our results demonstrate that both the frequency of responder individuals (75%–83%) and the intensity of SARS-CoV-2-specific responses were similar in all patients with IEI or IEI categories defined by the IUIS and the healthy control group, consistent with data previously reported in other IEI and CVID cohorts of patients, even when they were assessed by different *in vitro* methods and vaccine dosages (45–53). Since the literature regarding cellular response to COVID-19 vaccines in patients with IEI is conflicting, our results are in disagreement with the findings of other authors who have reported a declined SARS-CoV-2-specific T-cell response compared to healthy individuals (10, 11, 36, 54, 55). Lower anti-SARS-CoV-2 CD4^+^ T-cell memory responses correlate with the lack of generation of IgG-specific antibodies in patients with CVID (56, 57). CD8^+^ T cells are critical for viral control (12, 13), and a very recent study involving healthy subjects also determines a positive relationship between anti-SARS-CoV-2 AIM CD8^+^ T cells and neutralizing antibody responses (58), although CD8^+^ memory T-cell responses were not measurable even in the healthy control group of a previous study (57). Different vaccination strategies, time to evaluation, and methods to quantitate immunogenicity plus the heterogeneous nature of patients with IEI included in these studies may underlie such contradictory findings. Standardized methodologies to assess T cellular responses would be needed in order to reach clearer conclusions on the immune status against SARS-CoV-2 in vaccinated patients with IEI, and it may be advantageous to include CD8^+^ T-cell responses in future designs.

In contrast to SARS-CoV-2 stimulation, we found a significant decrease in the intensity of CMV-specific T-cell responses in the total group of patients with IEI and in our two major subgroups of PAD patients (CVID and IOFD) when compared to healthy donors. Despite CVID being considered a typical PAD, it is known that a subset of patients with CVID display additional features of cellular immunodeficiency, with viral infection as a clinically relevant hallmark (59, 60), which could explain the lower T-cell responses to latent CMV infection in our patients. The reasons why our patients with IEI could have a lower prevalence of CMV infection than our HCHC group remain unclear. HCHC with a median age of 50 years are not significantly younger than the entire IEI group with a median age of 54 years (not shown). Since a second peak in middle age of CMV primoinfection has been noted in developed countries (41), this peak might be reduced in our patients with IEI—mostly PAD—by some protection associated to IgRT or because of a decreased exposure in their IEI context when compared with healthcare workers.

Our results agree with the study of Hetemaki et al., who found impaired anti-CMV cellular functions by ELISpot in APECED (autoimmune polyendocrinopathy–candidiasis–ectodermal dystrophy) patients with IEI compared to healthy controls (61), and disagree with the findings of other authors who reported non-significant differences between the CD4^+^ T-cell responses of patients with CVID and healthy individuals as measured by IFN-γ secretion after CMV peptide stimulation (62). However, Raeiszadeh et al. described increased CMV-specific activated and terminally differentiated CD8^+^ T cells with functional responses (62). Since most patients in clinical immunology units have PAD, implementing the evaluation of cellular responses to CMV could help one to know the functionality of their T cells, as well as their susceptibility to CMV infection, or even whether they are reactivating CMV as detected by an increase in the T-cell response (63).

One of our patients with IEI showed undetectable SARS-CoV-2 CD4^+^ T-cell responses *in vitro* and repeated specific antibody generation failure, which were strikingly discordant with the strong CMV cellular and humoral responses observed throughout his entire follow-up, including five doses of mRNA COVID-19 vaccines (35). Of note, this particular patient has shown S-specific T-cell response after the sixth SARS-CoV-2 vaccine immunization (not shown) while remaining naïve to natural SARS-CoV-2 infection (by means of repeatedly negative home nasal swab antigen tests when he was suspected of having COVID-19). This case of ours, along with the impact of CMV after primary COVID-19 vaccination recently reported within the oldest healthy adults from a Dutch general population cohort, prompted us to study a possible association between CMV status and SARS-CoV-2 Spike-specific CD4^+^ T-cell immunogenicity in our entire cohort of patients with IEI. Overall, our results support the idea that variation in SARS-CoV-2 T-cell immune responses could be due to CMV infection.

How CMV infection might compromise the quality of immune protection after vaccination remains poorly understood. CMV-induced changes in the T-cell repertoire (64), the significant driving of major expansions of specific effector and memory T cells to keep this β-herpes virus in latency, and the increased expression of senescence-associated markers (18, 20, 23) could underlie the worse quality of memory CD4^+^ T-cell responses to neoantigens from SARS-CoV-2 observed in the entire group of patients with IEI or in patients with CVID. The lack of impact of CMV latency in our group of patients with IOFD could be related to the active replication of CMV infection. CMV viremia was not tested in our patients, but it has been recently reported to be positive and related to cellular dysregulation in 16% of patients with CVID (65, 66) and in none of the patients with IOFD included in a previous study (65). Our findings are in line with the fascinating study of Bowyer et al. (67), who also had the opportunity to evaluate the impact of CMV on vaccine-induced immune responses to another neoantigen—the Ebola glycoprotein—and found that the higher CMV seroprevalence in Africa was associated with reduced vaccine-induced responses to Ebola. There are other previous demonstrations of the ability of CMV to reduce immune system responses to more common immunizations, mainly derived from studies after influenza (68, 69) and tick-borne encephalitis vaccines (70). Controversial results have been reported in these investigations (68, 69, 71, 72), and a meta-analysis studying the influence of CMV serostatus on the humoral response to influenza vaccination has concluded with insufficient evidence (73).

The passage of time also has an inevitable role in immunosenescence, and age is another risk factor for poor cellular responses induced after vaccination (74, 75). There is also an increasing agreement that the impact of CMV can differ depending on the age of the host, with worse immune responses in older than in younger individuals (20). Therefore, we took into consideration age-associated immunosenescence as potentially responsible for a skewed repertoire with less effector T-cell functions (20, 76, 77). In our IEI group, multivariant analysis did not show a significant effect of age on the T-cell response to SARS-CoV-2 following two vaccination doses. Since 50% of our patients were under the age of 54 years, we hypothesize that their immunodeficient condition could be adding to CMV status with an equivalent effect as the age.

The cross-sectional design of the present study does not allow causal inferences or longitudinal assessment. Unfortunately, we were unable to compare *in vitro* antigen-specific CD4^+^ T-cell responses in those subgroups of non-PAD IEI made up of a small number of patients. For the same reason, some observations in our study may also be limited by the low representation of very young or elderly individuals. Further studies in different cohorts devoted to younger and older patients and healthy donors are needed in order to validate the data presented here; it would also be interesting to analyze the long-term impact of CMV infection on immunogenicity to subsequent COVID-19 immunizations and hybrid immunity.

In conclusion, here we have demonstrated the negative impact of latent CMV infection on immunogenicity after two doses of mRNA SARS-CoV-2 vaccines in selected forms of PAD patients like CVID. We emphasize the importance of understanding the immune status of CMV and tailoring booster strategies for vaccine-preventable diseases by using novel T-cell functional assays, not only for the vaccines mentioned above but also for other vaccines (i.e., herpes zoster vaccination), in immunocompromised patients.

## Data Availability

The raw data supporting the conclusions of this article will be made available by the authors, without undue reservation.

## Supplementary material

Supplementary Figure 1 Identification of antigen-specific CD4+ T lymphocytes following two doses of mRNA-COVID-19 vaccination through AIM assays. **(A)** Gating strategy of effector memory CD4+ T-cells (TEM) in a patient with inborn error of immunity (IEI) and a healthy control. The lymphocyte gate was defined based on forward scatter (FSC) and side scatter (SSC) characteristics of PMBC. Then, CD3^+^CD4^+^ helper T-cells were selected, and CD4^+^ TEM cells were identified by the absence of CD45RA and CD27 surface expression. **(B)** Illustrative density plots showing the TEM responses to CMV pp65 and spike-SARS-CoV-2 peptides in two representative patients with IEI (P-1 and P-2) and healthy controls (HCHC-1 and HCHC-2).
