# Supplementary figures and images for: Impact of CMV latency on T-cell responses to COVID-19 vaccination among predominantly antibody-deficient patients
Source: Front Immunol. 2025 Sep 30;16:1659259. doi: 10.3389/fimmu.2025.1659259 (PMC12518298; doi:10.3389/fimmu.2025.1659259)

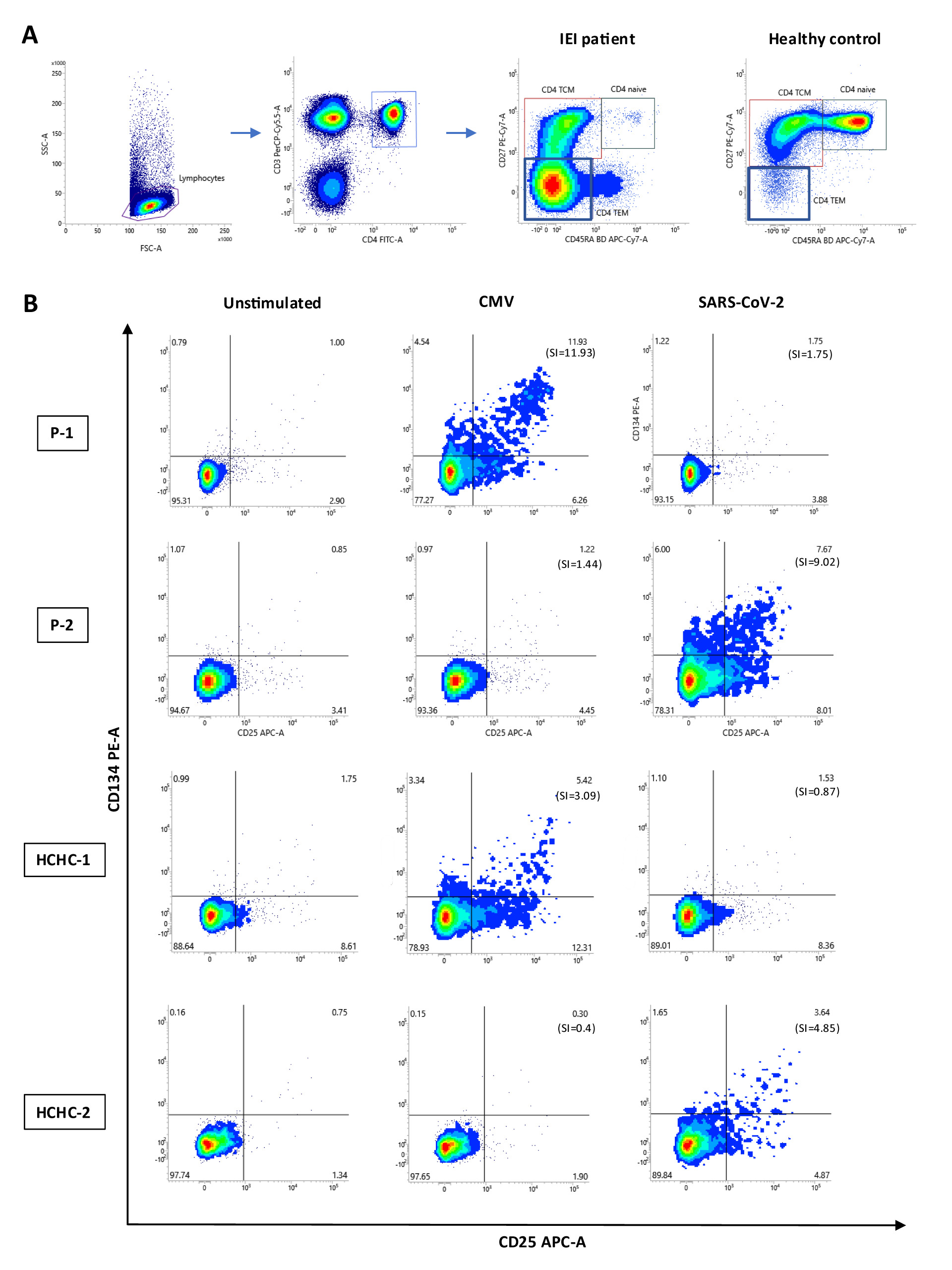

Supplement: Supplementary Figure 1 — Identification of antigen-specific CD4+ T lymphocytes following two doses of mRNA-COVID-19 vaccination through AIM assays. (A) Gating strategy of effector memory CD4+ T-cells (TEM) in a patient with inborn error of immunity (IEI) and a healthy control. The lymphocyte gate was defined based on forward scatter (FSC) and side scatter (SSC) characteristics of PMBC. Then, CD3+CD4+ helper T-cells were selected, and CD4+ TEM cells were identified by the absence of CD45RA and CD27 surface expression. (B) Illustrative density plots showing the TEM responses to CMV pp65 and spike-SARS-CoV-2 peptides in two representative patients with IEI (P-1 and P-2) and healthy controls (HCHC-1 and HCHC-2). [file Image1.jpg]
